# Editorial: Cancer Models

**DOI:** 10.3389/fonc.2018.00401

**Published:** 2018-10-03

**Authors:** Michael Breitenbach, Jens Hoffmann

**Affiliations:** ^1^Department of Biosciences, University of Salzburg, Salzburg, Salzburg, Austria; ^2^Experimental Pharmacology and Oncology Berlin-Buch GmbH, Berlin, Germany

**Keywords:** tumor models, PDX (patient-derived xenografts), tumor cell, Transgene mouse, 3D cell biology

Cancer is still a major concern for public health and is a cause of death while being psychologically the most dreaded disease. Until recent times, the diagnosis of a progressed form of cancer invariably meant that there was little chance for long-term survival. This notion has changed recently thanks to the tremendous efforts in cancer research and development (compared to other diseases) supported by enormous public and private financing. To illustrate this, just consider the “moonshot initiative” announced by US President Obama in 2016 (1).

Cancer research, like research in other diseases, highly depends on representative and reliable model systems. However, cancer is not the single molecule-defined tumor, but rather a heterogenic and highly variable complex of different individual diseases. This makes research on cancer highly difficult, expensive, and explains the enormous resources needed.

Looking back on more than 100 years of cancer research, the models used have changed constantly and extended permanently to all the new stages of drug discovery including target identification, lead structure optimization, tolerability, toxicity, biomarker discovery, and individual patient prediction. Today, the selection of the most appropriate model to best reflect the given tumor entity is one of the major challenges for the cancer researchers.

From the available volumes on the research topic “Cancer Models,” we collected original papers and review articles that addressed the topic of tumor modeling from perspectives such as molecular biology, biochemistry, microorganisms, cells, and organoids, fishes, animals, and xenografts, up to computational cancer models and patient data analysis.

Next-generation sequencing-based methods have recently revealed complex patterns of chromosomal aberrations, which are beyond explanation by classical models of karyotypic evolution. The term chromothripsis has been introduced to describe the phenomenon of temporarily and spatially confined genomic instability. This results in dramatic chromosomal rearrangements of segments of one or a few chromosomes. Simultaneously, misrepaired DNA double-strand breaks are causing another phenomenon called chromoplexy, which is characterized by the presence of chained translocations and interlinking deletion bridges in chromosomes.

Steininger et al. have been new models for the molecular biology underlying tumorigenesis. They developed a technology for genome-wide identification of chromosomal translocations based on the analysis of translocation-associated changes in spatial proximities of chromosome territories. Using the sequence data from a cutaneous T-cell lymphoma for genome-wide chromosome conformation capture (Hi-C), they fine-mapped chromosomal breakpoints and correlated them with gene-associated biological processes like transcription. As identification and functional analysis of chromosomal aberrations are basic for cancer research, such genome-wide high-resolution analyses of structural chromosomal aberrations will gain increasing importance.

In this light, Tosato et al. describe a model for the functional validation of gene translocations in yeast models. Genetic fusions between NUP145 and TOP2 in the human orthologs are associated with acute myeloid leukemia (AML). This translocant yeast strain can be used as a model to provide new grounds for a possible involvement of p53 in cell transformation toward AML.

A better understanding of tumor cell biology has led to the development of new model systems for certain functional processes that are related to the development and growth of cancer.

Some of these new methods and models are reviewed in the chapters of Schosserer et al., Lorz et al., Valenzuela et al., and Auer et al. In the individual cancers, the mechanisms for tumor hypoxia and senescence and cytoskeletal organization vary. Thus, specialized pharmacological agents need to be applied after characterizing a specific tumor with new and advanced biochemical methods. This includes tumor neo-antigens, specific ways of deregulation in the tumor, genomic sequencing of the tumor and the patient, and various omics methods applied to the tumor material and to patient-derived xenografts. Precision medicine means that a large amount of the data just mentioned is now being used to construct a pathway model of the individual tumor with the help of new bioinformatics methods, often called “big data” or “data science.”

A very important and relevant topic in cancer research in the twenty-first century is the role of cellular senescence and dormancy in the etiology of tumor formation and of therapy resistance of tumors. No fewer than three of the chapters published in the present volume deal with this topic: Schosserer et al., Yadav et al., and Valenzuela et al. Cellular senescence of cultured human cells was discovered by Hayflick (2) and termed senescence. Ever since that discovery, the “Hayflick phenomenon” or “Hayflick aging” was discussed as a possible cause of organismic aging. For a long time, it was unclear if Hayflick aging is perhaps an artifact of cell culture–because lowering the oxygen partial pressure during cell culture to the low levels occurring in peripheral tissue can indeed substantially increase the lifespan of a cell. Biochemical markers of senescence were discovered, such as the senescence-associated beta-galactosidase (3) and the senescence-associated secretory phenotype (4) and relatively recently, it was shown beyond doubt that senescent cells do occur *in vivo* in old individuals and constitute one cause of organismic aging. The latter fact was shown by eliminating the senescent cells by selectively inducing apoptosis of these cells without general induction of apoptosis in the mouse. Compounds able to do this are called senolytics (5). Even naturally aged mice regained juvenile organ characteristics by this treatment, for instance in the kidney (4, 6). Senescent cells are found in tumors (7) and, quite frequently, chemotherapy spares those cells and leads to an often deadly relapse of the disease. The senescent cells can, through release of cytokines (the senescent cell secretome), induce cancerous growth in surrounding cells. An important aim for cancer therapy would be not only to kill the fast dividing cancer cells, but also to kill, like in the experiments of Baar et al. (6), the senescent cells contained in the tumors. This relatively new development completely replaces the old theory that senescence was primarily “invented” by evolution to avoid the formation of tumors (8).

The importance of cellular senescence in cancer biology is further stressed in the hypothesis and theory chapter by Valenzuela et al. These authors explain the role of the senescence associated secretory phenotype (SASP) in the MET and in the formation of metastases. In this process, which is intimately connected also to aging and the permanent low level of inflammation in aged individuals, platelets play a central role, which was not studied intensively in the past.

While classical 2D cell culture models have been the mainstay for cancer research, current limitations of cell cultures are now partially overcome by 3D cell cultures that allow a more accurate mimicry of the native cancer tissue, since they preserve cellular heterogeneity and restore some of the specific biochemical and morphological features similar to the corresponding tissue *in vivo*. This is a fundamental advantage, because both morphology and cell-environment crosstalk strongly influence gene expression and, therefore, cell behavior.

They have gained an important role for research on tumor micro-environmental regulation of proliferation, invasion, cell-cell interaction and metabolism, used between cellular monolayers and animal models. The 3D cell cultures have served as a model for a variety of experimental therapy studies using radiotherapy, chemotherapy, as well as cell- and antibody-based immunotherapy. The current developments in 3D cultures are thoroughly reviewed by Gaebler et al. in this volume.

Modeling of tumor disease in animals goes back to more than 100 years to the first experiments with the transplantation of syngeneic tumors in inbred mouse strains (9). These models are mainly replaced by “human” tumor models, the pros and cons of which have been described in more details by Klinghammer et al. (10).

A relatively new animal model, in between cell culture and mammalian models, is based on the zebrafish (*Danio rerio*). The zebrafish model, treated here in the chapter written by Kirchberger et al., offers the important advantage of extracorporeal fertilization, and a very well-researched translucent embryo, as well as short generation time and a well-developed genetical toolbox. All this makes this little fish an ideal model system for studying development. Cancer, after all, is a deviation from normal development, caused by aberrations in gene expression. In a zebrafish melanoma model, it could be shown that at the base of cancer formation is the de-differentiation of epithelial cells to form embryonic neural crest cells (11). Due to the small size, short generation time, and extracorporeal development of the fish, it was possible to perform several large-scale screens (in a typical case more than 26,000 compounds) of chemical libraries to find compounds that could inhibit specific cancers or metastasis in transgenic fish containing patient-derived xenografts (12).

Using mouse models to study human tumor growth dates back to the late 1960s. Development of immune-deficient mouse strains like nude or severe combined immunodeficiency (SCID) allowed a continuous improvement of these models, which can be used for xenotransplanting human cell lines or human tumors directly as patient-derived xenotransplants (=PDX) or transplant human cell lines [for review see Behrens et al. (13)].

Two further animal models are treated in separate chapters in this volume. As we know now, it is in nearly all cases impossible to study the very early stages of the disease in humans due to the very long time interval between a primary causative mutational event and the first detection of a tumor in the clinic. However, it is possible to look at these early stages of tumor development in certain highly sophisticated transgenic mouse models, which allow the researchers to induce expression of an oncogenic mutation in an otherwise healthy animal at a specific time and in a specific organ (see the chapter by Lampreht-Tratar et al. on “transgenic mouse models in cancer research”).

Genomically, mice and humans are reasonably closely related as about 80% of all protein coding genes of humans share closely related orthologs with the mouse, including most of the recognized genes involved in cancer. What makes the mouse model so attractive is the short generation time (10 weeks) and average life expectancy (2.5 years in commonly used laboratory mice), the possibilities for reverse genetics, meaning the possibility to introduce at will nearly all genetic alterations desired to study cancer biology, and the frequent occurrence of cancer even in the absence of cancerogenic agents, with an exponential increase in old age, like in humans. Of course, not all questions posed by human cancers can be answered in one animal model.

The domestic pig is an additional new animal model for cancer, which is treated in detail in the chapter on the Oncoming Cancer Model by Schachtschneider et al. in the present volume. This chapter very well describes both the differences and shortcomings of the mouse cancer model and the surprising similarities between the human and pig cancer biology. We name just a few important experimental findings pertaining to the comparison of these cancer models. First, the loss of heterozygosity (LOH) mechanism, which is very common in human cancers, in particular in adenomatous polyposis coli (APC) leading to colon cancer appears to be very infrequent in mice. Second, the cytochrome P450 system of mice is very different from the human system, making a detailed comparison of the cancerogenic potential of xenobiotics is difficult in the mouse. On the other hand, the cytochrome P450 system is very similar in swine and humans. Liver cirrhosis and hepatocellular carcinoma (HCC) can be efficiently induced in pigs and the experimental therapeutical possibilities (surgical as well as chemotherapeutic) can be well studied in the pig. Generally, the large size of the pig make it easier to study therapeutic approaches, typical comorbidities and the interaction of cancers with the microbiome are similar to the ones found in human patients.

So it appears that the three animal model systems presented here together with several others not mentioned presently can all lead the way to answering different open questions in cancer biology complementing each other. In all three animals, the complete sequence of the genome is known and the techniques of genetic manipulation needed for cancer research are well developed. These techniques include reverse genetics including the modern CRISPR/Cas9 system in several forms, the creation of conditional mutations in cancer-relevant genes, and the cloning of animals by somatic cell nuclear transfer (SCNT).

We just have to mention that during the last 10 years, the biggest impact and improvement in cancer survival were without any doubt the introduction of new ways to raise the patient's immune defense against cancer cells as a new form of therapy. This was achieved not only through the newly developed humanized antibodies that recognize cancer cells through binding to neo-antigens, but also due to more subtle interference with the natural regulators of the immune system, for instance IL2, aiming at the induction of apoptosis of tumor cells without imposing a general cytostatic load onto the patient. Spectacular examples of cancers where 5-year survival was extremely low and a complete cure is now possible include certain forms of melanoma (14). Models for tumor immunology are extremely challenging and will be addressed in a separate research topic.

In our times, literature data are becoming more and more important for cancer research. New mathematical models analyzing literature data can be used to identify or validate new tumor targets. For example, hypoxia is an important topic in cancer research that was investigated continuously over several decades, starting with the seminal papers of Warburg (15). Rausch et al. summarize the results of 18 publications dealing with the correlation of breast cancer incidence with adipocytes and severe obesity. Adipocytes are a source of several hormones like adiponectin, which is positively correlated with breast cancer incidence and progression. By downstream signaling via HIF-1apha, adiponectin can contribute to the formation of the breast tumors.

In the final two chapters of this volume, the authors are dealing with theoretical and mathematical models developed in cancer research. Ogilvie et al. in their “Perspective” contribution present meta-theoretical considerations on mathematical models in cancer research and treat questions like these: What are the basic and most important prerequisites which are to be met, if a theoretical model shall benefit cancer research? Which are the results that were produced up to now? What has been modeled and what is currently impossible to describe meaningfully in a theoretical model? This chapter deals primarily with the possibility (or currently “impossibility”) to use bioinformatics models to create a truly personalized medicine (16, 17). This would be based on the ever increasing datasets provided by the “omics” methods as applied to tumor material, the genome of the tumor and of the patient, and the metabolic pathways revealed by metabolomics. Lorz et al., in their chapter, present original research done with a mathematical model based on partial differential equations dealing with growth of cancer cell lines *in vitro* under the influence of variable parameters like initial cell density and, most importantly, concentrations of cell cycle inhibitors (anti-mitotic agents). During the *in silico* experiment, the cells populate three different compartments: proliferation (active cell division), quiescence (blocked cell division and survival), and apoptosis (programmed cell death). The simulations correctly predict under which conditions the actively dividing tumor cells will die out and the kinetics of the transitions between the three compartments. Of note, the quiescent (dormant) state, corresponding to a reversible cell cycle arrest, is a necessary component of the model. It has been covered in three other chapters in the present book (see above) and plays a central role in contemporary cancer research.

## Author contributions

All authors listed have made a substantial, direct and intellectual contribution to the work, and approved it for publication.

### Conflict of interest statement

The authors declare that the research was conducted in the absence of any commercial or financial relationships that could be construed as a potential conflict of interest.
